# Comparative genomics of grass EST libraries reveals previously uncharacterized splicing events in crop plants

**DOI:** 10.1186/s12870-015-0431-7

**Published:** 2015-02-05

**Authors:** Trees-Juen Chuang, Min-Yu Yang, Chuang-Chieh Lin, Ping-Hung Hsieh, Li-Yuan Hung

**Affiliations:** Genomics Research Center, Academia Sinica, Taipei, 11529 Taiwan

**Keywords:** Crop plants, Alternative splicing, Plant transcriptome evolution, Evolutionary rate, Comparative genomics, Bioinformatics

## Abstract

**Background:**

Crop plants such as rice, maize and sorghum play economically-important roles as main sources of food, fuel, and animal feed. However, current genome annotations of crop plants still suffer false-positive predictions; a more comprehensive registry of alternative splicing (AS) events is also in demand. Comparative genomics of crop plants is largely unexplored.

**Results:**

We performed a large-scale comparative analysis (ExonFinder) of the expressed sequence tag (EST) library from nine grass plants against three crop genomes (rice, maize, and sorghum) and identified 2,879 previously-unannotated exons (i.e., novel exons) in the three crops. We validated 81% of the tested exons by RT-PCR-sequencing, supporting the effectiveness of our *in silico* strategy. Evolutionary analysis reveals that the novel exons, comparing with their flanking annotated ones, are generally under weaker selection pressure at the protein level, but under stronger pressure at the RNA level, suggesting that most of the novel exons also represent novel alternatively spliced variants (ASVs). However, we also observed the consistency of evolutionary rates between certain novel exons and their flanking exons, which provided further evidence of their co-occurrence in the transcripts, suggesting that previously-annotated isoforms might be subject to erroneous predictions. Our validation showed that 54% of the tested genes expressed the newly-identified isoforms that contained the novel exons, rather than the previously-annotated isoforms that excluded them. The consistent results were steadily observed across cultivated (*Oryza sativa* and *O. glaberrima*) and wild (*O. rufipogon* and *O. nivara*) rice species, asserting the necessity of our curation of the crop genome annotations. Our comparative analyses also inferred the common ancestral transcriptome of grass plants and gain- and loss-of-ASV events.

**Conclusions:**

We have reannotated the rice, maize, and sorghum genomes, and showed that evolutionary rates might serve as an indicator for determining whether the identified exons were alternatively spliced. This study not only presents an effective *in silico* strategy for the improvement of plant annotations, but also provides further insights into the role of AS events in the evolution and domestication of crop plants. ExonFinder and the novel exons/ASVs identified are publicly accessible at http://exonfinder.sourceforge.net/.

**Electronic supplementary material:**

The online version of this article (doi:10.1186/s12870-015-0431-7) contains supplementary material, which is available to authorized users.

## Background

Alternative splicing (AS) is a major post-transcriptional mechanism for producing multiple isoforms from the same precursor mRNA (pre-mRNA), thereby increasing the complexity of the transcriptome/proteome. AS is widespread in eukaryotes, and it has been suggested that over 95% of genes in human are alternatively spliced [1,2]. In contrast, 30% ~ 60% of genes in *Arabidopsis* or rice have been identified to undergo AS [3-11]. AS appears to be relatively less prevalent in plants than in mammals, but this may in part be due to limited detection of alternatively spliced variants (ASVs) in plants.

AS has been demonstrated to be involved in various biological functions [12-16] such as spatio-temporal regulation [17-20], disease resistance [21], and photosynthesis [22,23]. ASVs occur in both coding sequences (CDSs) and untranslated regions (UTRs). ASVs in CDSs can have influences on protein structure, subcellular localization, protein stability, post-translational modifications, enzymatic activity, and protein-protein interaction networks [24-26]. On the other hand, ASVs in 5′ UTRs (3′ UTRs) may include/exclude upstream open reading frames (premature termination codons), thereby altering translational stability/efficiency (nonsense-mediated decay pathway) [14,27]. Even so, a considerable number of ASVs are functionally irrelevant, or merely by-products during RNA splicing [28,29]. It remains challenging to determine whether an ASV is functionally important [30-33], not to mention that AS is less characterized in plants than in mammals, and that most plant ASVs have unknown functional consequences [10], but also that some of computationally-annotated genes/transcripts are subject to erroneous prediction. Although much effort to annotate plant transcripts produces several prominent databases [34-39], there still lacks an effective strategy to make use of public resources (e.g., EST traces) for better annotation of ASVs and accurate identification of novel isoforms in plant genomes.

In terms of molecular evolution, alternatively spliced exons and constitutively spliced exons are known to be under different evolutionary pressures. Previous studies reported that alternatively spliced exons tend to have higher nonsynonymous substitution rates (*dn*) and nonsynonymous-synonymous substitution rates (*dn/ds*) than constitutively spliced ones, indicating faster protein-level evolution in the former [40-47]. On the other hand, alternatively spliced exons were observed to have lower *ds* values than constitutively spliced ones due to the elevated synonymous rate in the latter [47]. This suggests that constitutively spliced exons are subject to weaker selection pressure than alternatively spliced ones at the RNA level. Therefore, the differences in evolutionary patterns may serve as an indicator to distinguish between these two types of exons.

In this study, we aimed to update the annotations of three crop plants, namely rice (*Oryza sativa*), maize (*Zea mays*), and sorghum (*Sorghum bicolor*). We designed a pipeline, ExonFinder, for the identification of novel exons/ASVs based on comparative genomics of the EST libraries of nine grass plants, including barley (*Hordeum vulgare*), maize, meadow ryegrass (*Festuca pratensis*), purple false brome (*Brachypodium distachyon*), rice, sorghum, sugarcane (*Saccharum officinarum*), switchgrass (*Panicum virgatum*), and wheat (*Triticum aestivum*). Such analysis resulted in the identification of a total of 2,963 ASV events (including cassette exons and retained introns) in rice, maize, and sorghum, with 2,879 novel exons that were cross-species conserved but not supported by prior Ensembl annotation or EST evidence from the same species. Evolutionary analysis reveals that though the novel exons are generally under more relaxed selection pressure than their flanking ones, some of them evolve at a similar evolutionary rate with their flanking exons. We reasoned that some of the previously-annotated isoforms that excluded the newly-identified exons may be subject to erroneous prediction. To test this possibility, we randomly selected rice exons of this kind, performed RT-PCR-sequencing, and found that over half (54%) of previously-annotated isoforms that excluded the novel exons were not detected in the same setting. The consistent results were observed in three rice cultivars (i.e., *O. sativa* L. ssp*. Indica* cv. 93-11, *O. sativa* L*.* ssp. *japonica* cv. Nipponbare, and *O. glaberrima*) and two wild rice species (i.e., *O. rufipogon* and *O. nivara*). Finally, we also discussed the functional potential of selected ASVs through the lens of evolution.

## Results

### Identification of novel exons in rice, maize, and sorghum

**Figure 1:**
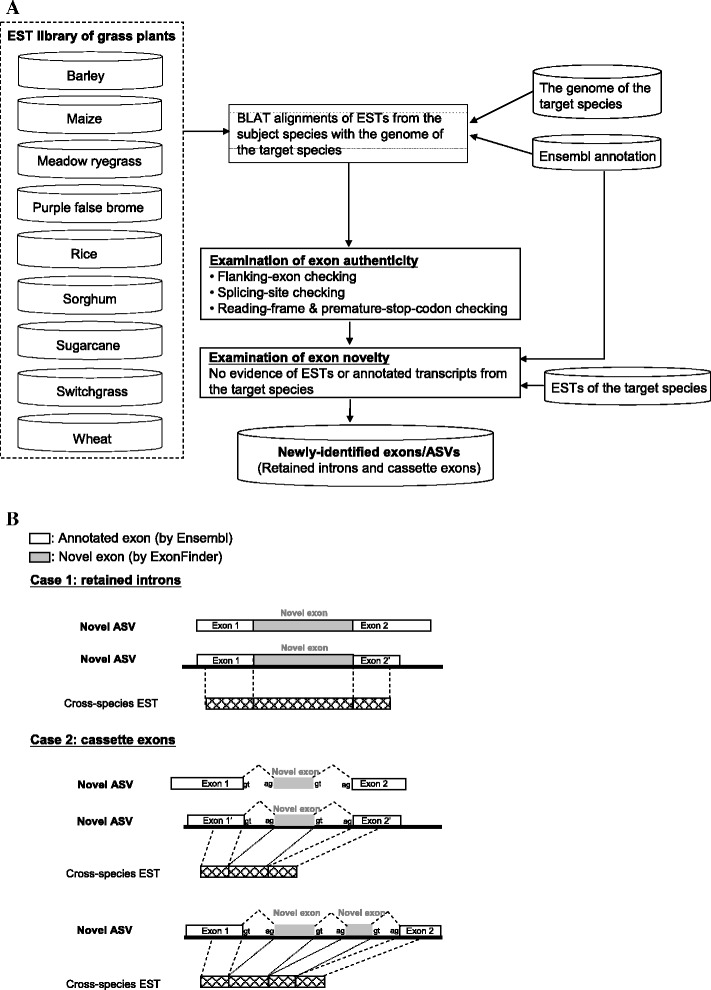
**The ExonFinder process. (A)** Flowchart of the identification of novel exons by ExonFinder. **(B)** Examples of newly-identified exons and ASVs, including retained introns (Case 1) and cassette exons (Case 2).

We introduced an *in silico* pipeline, ExonFinder, to identify previously unannotated exons/ASVs in target species (i.e., rice, maize, and sorghum) by comparative analysis of the EST library of non-target (designated as “subject”) species against the genome of target species (Table 1 and Figure 1A). To achieve a better quality of cross-species alignment, we only considered grass plants in this study (Table 1). We supposed that the novel exons also represented novel AS events, since they were absent from known transcripts of the target species (Methods). ExonFinder identifies two types of novel exons: cassette exons and retained introns (Figure 1B). Authenticity and novelty of exons were considered through the following procedures. To eliminate false positives from accidental matches, we only considered EST matches that satisfied the following criteria: (1) a proper exon and its flanking exons must overlap with the same Ensembl-annotated transcript; (2) a proper cassette exon must be flanked by canonical splicing sites at its both ends; and (3) a proper exon that locates within CDS must not change the reading frame and must not result in any premature stop codon. Of note, Exonfinder also identifies novel cassette exons flanked by non-canonical splicing sites (Methods), although we only considered those flanked by canonical splicing sites for accuracy in the following analysis. To distinguish novel exons from currently-characterized exons, we removed the exons that were supported by Ensembl’s annotation or EST traces from the target species (Methods). Of note, for each newly-identified transcript (or novel ASV), it must include at least one full-length novel exon and the flanking exons’ segments of the novel exon(s) (Figure 1B). It is possible for a novel exon to be assigned to more than one novel ASV, in the case of uncertain boundaries of the flanking exons (Figure 1B). In addition, a novel ASV may also contain multiple novel exons (Case 2; Figure 1B). Consequently, we used ExonFinder to identify a total of 382 (381), 1,245 (1,150), and 1,336 (1,348) novel ASVs (novel exons) in rice, maize, and sorghum, respectively (Table 2 and Additional file 1).

**Table 1:** **Summary of EST traces used in this study**

**Plant species**	**Abbreviation**	**UniGene version**	**Number of EST traces**
Barley (*Hordeum vulgare*)	Hv	Build #59	517,604
Maize (*Zea mays*)	Zm	Build #81	1,705,606
Meadow ryegrass (*Festuca pratensis*)	Fp	Build #1	60,845
Purple false brome (*Brachypodium distachyon*)	Bd	Build #2	113,694
Rice (*Oryza sativa*)	Os	Build#86	1,202,546
Sorghum (*Sorghum bicolor*)	Sb	Build #30	199,401
Sugarcane (*Saccharum officinarum*)	Sof	Build #15	220,997
Switchgrass (*Panicum virgatum*)	Pv	Build #3	505,999
Wheat (*Triticum aestivum*)	Ta	Build #59	1,050,213

**Table 2:** **Number of newly-identified exons/ASVs (including cassette exons and retained introns) in rice, maize, and sorghum**

		**Newly-identified exons (ASVs)**		
**Species**	**Genomic type**	**Cassette**	**Retained intron**	**Total**
**Rice**	5′-UTR	36 (42)	8 (9)	44 (51)
	CDS	214 (216)	70 (70)	284 (286)
	3′-UTR	47 (38)	6 (7)	53 (45)
	Total	297 (296)	84 (86)	381 (382)
**Maize**	5′-UTR	272 (324)	95 (104)	367 (428)
	CDS	364 (367)	83 (85)	447 (452)
	3′-UTR	202 (209)	134 (156)	336 (365)
	Total	838 (900)	312 (345)	1,150 (1,245)
**Sorghum**	5′-UTR	242 (230)	53 (55)	295 (279)
	CDS	672 (684)	224 (227)	896 (913)
	3′-UTR	98 (78)	59 (62)	157 (140)
	Total	1,012 (992)	336 (344)	1,348 (1,336)

### Basic properties of the newly-identified exons/ASVs

As shown in Table 3, most of the identified exons/ASVs were supported by multiple EST traces, indicating these isoforms might not be rare. In addition, 14% ~ 30% of identified exons/ASVs were supported by EST traces from at least two non-target species, implying that they were widely expressed in grass plants. Since evolutionary conservation implies functional importance [33,48], these exons/ASVs may play an important role in grass plants, rather than random by-products during RNA splicing. Furthermore, the average length (~100 bp) of the novel cassette exons (Table 3) were considerably shorter than the average exon length (250 ~ 300 bp) of previously-annotated exons in rice, maize, and sorghum [3,26,49-51], reflecting a previous observation that conserved alternatively spliced exons tend to be shorter than non-conserved ones [48]. Next, we retrieved pure introns (i.e., constitutive introns; the Ensembl-annotated introns that do not contain any ExonFinder/Ensemble-identified alternatively spliced exons, and are flanked by two Ensemble-annotated constitutively spliced exons), and demonstrated that the average and median lengths of pure introns were significantly shorter than other known introns that contain the novel cassette exons (*P* value < 10^−6^ by the two-tailed *t*-test and Wilcoxon rank-sum test). This trends hold well across rice, maize, and sorghum, consistent with a previous observation that cassette exons tend to be flanked by longer introns than constitutively spliced exons [52].

**Table 3:** **General properties of the newly-identified exons/ASVs**

	**Rice**	**Maize**	**Sorghum**
Average number of supported EST traces (for cassette exons)	4.60	2.76	4.70
Average number of supported EST traces (for retained introns)	8.17	1.90	4.60
Percentage of the identified ASVs with EST evidence from ≥ 2 subject species	29.84	14.13	26.06
Average/median length of novel cassette exons (bp)	104.64/87	102.36/81	98.76/84
Average/median length of novel retained introns (bp)	105.40/81	108.72/92	112.19/93
Average/median length of the Ensembl-annotated introns that contain the novel cassette exons (bp)	1057.90/648	1665.04/556.5	932.09/547
Average/median length of pure introns (bp)	381.09/163	530.57/139	441.51/143
Significance test^*^	*P* < 10^−6^; *P* < 10^−15^	*P* < 10^−7^; *P* < 10^−15^	*P* < 10^−15^; *P* < 10^−15^

^*^Differences between the average/median lengths of previously-annotated introns that contain the newly-identified cassette events and those of pure introns were examined using the two-tailed *t*-test and Wilcoxon rank-sum test, respectively.

**Figure 2:**
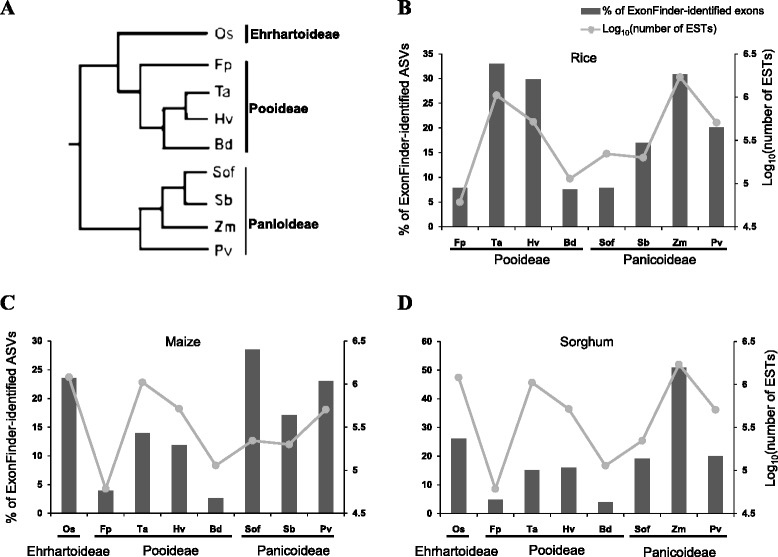
**Comparative analysis of the AS events extracted from different subject species. (A)** Phylogeny of the nine grass plants examined in this study [53,54]. These plants can be classified into three groups: Ehrhartoideae, Pooideae, and Panicoideae. **(B-D)** Comparison between the percentages of AS events identified from EST traces and the numbers of available EST traces of each subject species for Exonfinder identifications in three target species: rice **(B)**, maize **(C)**, and sorghum **(D)**. Os, rice; Fp, meadow ryegrass; Ta, wheat; Hv, Barley; Bd, purple false brome; Sof, sugarcane; Sb, sorghum; Zm, maize; Pv, switchgrass.

We found that ExonFinder identified much more novel ASVs in maize and sorghum (both >1,000 ASVs) than in rice (382 ASVs). This was not unexpected, as the annotation of rice genome was more comprehensive than those of maize and sorghum. In addition, the number of exons identified by ExonFinder is related not only to the number of available EST traces but also to the level of divergence between the target and subject species. According to earlier phylogenetic analyses [53,54], the nine grass plants examined in this study can be classified into three groups: Ehrhartoideae (including rice), Pooideae (including purple false brome, meadow ryegrass, barley, and wheat), and Panicoideae (including switchgrass, maize, sorghum, and sugarcane), indicating a closer relationship between Ehrhartoideae and Pooideae (Figure 2A). In rice, the percentages of novel ASVs identified from non-rice grass plants were generally positively correlated with the quantities of Pooideae and Panicoideae EST traces, respectively (Figure 2B). However, the percentages of novel ASVs identified from Pooideae EST traces tended to be higher than those identified from Panicoideae EST traces. This tendency might reflect that the level of divergence between Ehrhartoideae (i.e., rice) and Pooideae is lower than that between Ehrhartoideae and Panicoideae (Figure 2A). For example, although the number of EST traces of maize (>1.7 million) is larger than that of wheat (~1 million), both data sets were used to identify similar percentages of novel exons in rice (Figure 2B). On the other hand, ExonFinder using Pooideae EST traces tended to identify fewer novel maize/sorghum exons (both of which belong to Panicoideae) than that using Panicoideae EST traces, even though EST traces from Pooideae (e.g., wheat) are about five times more than those from Panicoideae (e.g., sorghum in Figure 2C and sugarcane in Figure 2D). This indicates that ExonFinder is particularly powerful in the identification of novel exons/ASVs in poorly annotated species by using closely related species with abundant EST traces.

### Newly-identified exons tend to have higher *dn* values and lower *ds* values than their flanking exons

**Figure 3:**
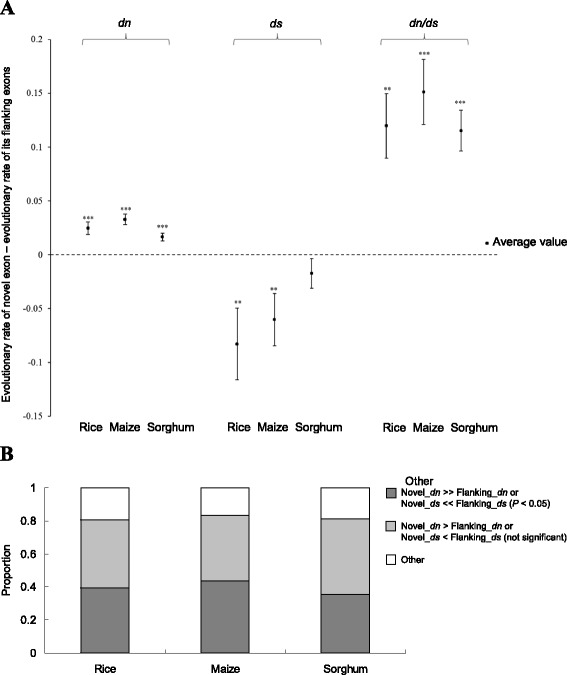
**Evolutionary analysis of the newly-identified exons and their flanking exons. (A)** Comparisons of evolutionary rates (*dn*, *ds*, and *dn*/*ds*) between the newly-identified exons and their flanking exons. Statistical significance was estimated by the paired two-tailed Wilcoxon signed rank-sum test. **P < 0.01 and ***P < 0.001. Error bars represent the standard errors of the means. **(B)** Proportions of newly-identified ASVs with and without significant differences in evolutionary rates between the novel exons and their flanking exons (*P* < 0.05 by the two-tailed Fisher’s exact test; Methods). Novel_*dn* and Novel_*ds* represent the *dn* and *ds* values of the novel exons; Flanking_*dn* and Flanking_*ds* represent the *dn* and *ds* values of their flanking exons, respectively.

To investigate the selection pressures imposed on the novel exons identified by comparative analysis of cross-species EST libraries, we calculated the evolutionary rates (*dn*, *ds*, and *dn/ds*) based on the alignments between the identified ASVs (including the novel exons and their flanking exons) in the target species and their corresponding EST sequences in the subject species (Methods). Since the novel exons are absent in the annotation (i.e., Ensembl annotation) of the target species, the inclusion level (the fraction of a gene’s transcript isoforms that include a specific exon [55]) should be lower for the novel exons than for their corresponding flanking exons. Previous studies have demonstrated that alternatively spliced exons have higher *dn* and *dn/ds* values, but lower *ds* values, than constitutively spliced exons, and that the inclusion level of exons is negatively correlated with *dn* and *dn/ds* values, but positively correlated with *ds* values [44,47,56]. Therefore, we reasoned that the novel exons should exhibit higher *dn* and *dn/ds* values, but lower *ds* values, than their corresponding flanking exons. To test this hypothesis, we concatenated the flanking exons of each novel exon, and then calculated the evolutionary rates of the novel exon and its flanking exons, respectively (Methods). After that, we calculated the differences of *dn*, *ds*, and *dn/ds* values between each novel exon and its corresponding concatenated flanking exons. As expected, the differences in average evolutionary rates between novel exons and their flanking exons were higher than zero for *dn* and *dn/ds*, but lower than zero for *ds* (Figure 3A), indicating that the novel exons had higher *dn* and *dn/ds* values, but lower *ds* values, than their flanking exons. This result suggested that the novel exons were subjected to weaker selection pressure than their flanking exons at the protein level (*dn* and *dn/ds*), but the trend was reversed at the RNA level (*ds*), consistent with our hypothesis.

Interestingly, although the trend that the majority of novel exons (~80%) have higher *dn* values or lower *ds* values than their corresponding flanking exons was observed in rice, maize, and sorghum, only less than 50% of cases showed significant differences in *dn* or *ds* between these two types of exons (Methods) (Figure 3B). In other words, a considerable proportion of novel exons do not exhibit significant difference in evolutionary patterns as compared to their flanking exons. There are two possible scenarios for this consequence. First, the novel exon also represents a novel AS events. There may be some undetected transcript isoforms that include the novel exon, but exclude one or two of their flanking exons, resulting in the inclusion level of the novel exon being higher than or equal to those of its flanking exons. Second, the novel exon does not represent an AS event (in fact, it is a constitutively spliced exon), while the previously-annotated one that excludes the novel exon may be subject to erroneous prediction. Relatively, it is more important to examine these potentially erroneous predictions.

### Certain previously-annotated isoforms remain non-evident by the existing transcript sequences

**Figure 4:**
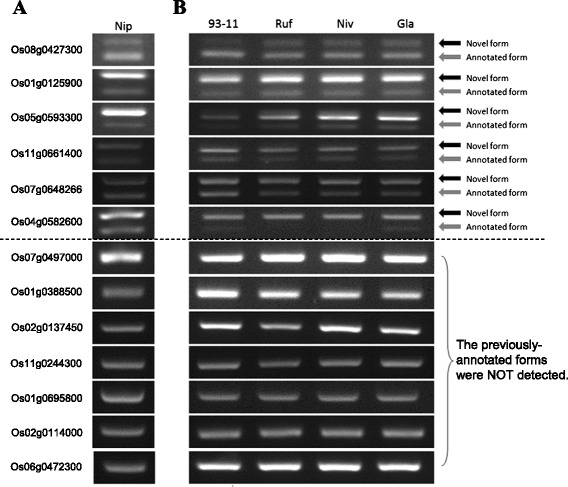
**Experimental validations of the newly-identified exons/ASVs.** Shown in the figure are. RT-PCR products of the newly-identified isoforms that include the novel exons and the previously-annotated isoforms that exclude the novel exons in **(A)** *O. sativa* L. ssp. *japonica* cv. Nipponbare (designated as “Nip”) and **(B)** *O. sativa* L. ssp. *indica* cv. 93-11 (designated as “93-11”), *O. rufipogon* (designated as “Ruf”), *O. nivara* (designated as “Niv”), and *O. glaberrima* (designated as “Gla”). The black and gray arrows represent the newly-identified and previously-annotated isoforms, respectively.

Taking rice as example, we then proceeded to confirm the authenticity of the newly-identified ASVs (i.e., the isoforms that include the novel exons and their flanking exons) and the previously-annotated ASVs (i.e., the isoforms that exclude the novel exons). Since the novel exons/ASVs identified here were based on the Ensembl annotation, we randomly selected 16 newly-identified ASVs and performed RT-PCR-sequencing experiments to examine their authenticity on a rice cultivar (i.e., *O. sativa* L*.* ssp. *japonica* cv. Nipponbare; Methods). The result showed that 13 of them (81%) were detected in *japonica* (Figure 4A and Additional file 2), supporting the effectiveness of ExonFinder. Intriguingly, while 13 novel AS isoforms were experimentally validated, more than half (54%; 7/13) of their previously-annotated isoforms were not detected (Figure 4A). We examined the alignments between rice EST traces (NCBI UniGene Database; Table 1) and the reference genome, and confirmed that no rice EST supported these previously-annotated isoforms. We further BLAST-aligned these previously-annotated transcript isoforms against the NCBI non-redundant database (Oct. 2014) and showed the absences of their homologous expressed sequences within other grass species. These results indicated that the previously-annotated isoforms were likely to be false positives. However, we cannot completely eliminate the possibility that these transcript isoforms are just absent in *japonica*, but are present in other cultivated or wild rice. To test this possibility, we attempted to detect these 13 newly-identified ASVs and their previously-annotated ASVs in other two cultivars (i.e., *O. sativa* L. ssp. *indica* cv. 93-11 and *O. glaberrima*) and two wild species (i.e., *O. rufipogon* and *O. nivara*) (Methods). Our results revealed that the 13 novel isoforms were steadily detected in all of the rice species examined, but the previously-annotated isoforms that were not detected in *japonica* were also absent in other rice species examined (Figure 4B). These results support that certain previously-annotated ASVs may be subject to erroneous prediction. In fact, except for Os06g0472300, all the previously-annotated isoforms that were not detected in our experiments have not included in the mostly updated version of the Ensembl annotation (Release 23). Of note, the three newly-identified ASVs that could not be detected in *japonica* were also absent in the other rice species examined (Additional file 2). Although it is possible that these exons might be lost in rice and became pure introns during evolution, we observed that two of them (Os04g28460 and Os11g34120) had a *dn/ds* ratio significantly smaller than 1 (both *P* values < 0.05 by the Fisher’s exact test). This indicates that these two newly-identified exons are subject to much stronger selective constrains on nonsynonymous changes than on synonymous ones [57-59], suggesting that they are more likely to be protein-coding exons.

Of the 13 experimentally-confirmed novel exons, 12 locate within CDS regions (Additional file 2). We observed that five exhibited significantly higher *dn* values or significantly lower *ds* values than their flanking exons, four of which were validated to be alternatively spliced (Figure 4 and Additional file 2). In contrast, the novel exons that exhibited neither higher *dn* values nor lower *ds* values than their flanking exons were not validated to be alternatively spliced (Additional file 2). This observation is consistent with the overall trend towards higher *dn* and lower *ds* values in alternatively spliced (or rarely utilized) exons as compared to constitutively spliced (or commonly utilized) exons, further suggesting that our evolutionary analysis is helpful for determining whether a newly-identified exon undergoes AS.

### Implications of newly-identified ASVs for evolutionary studies

**Figure 5:**
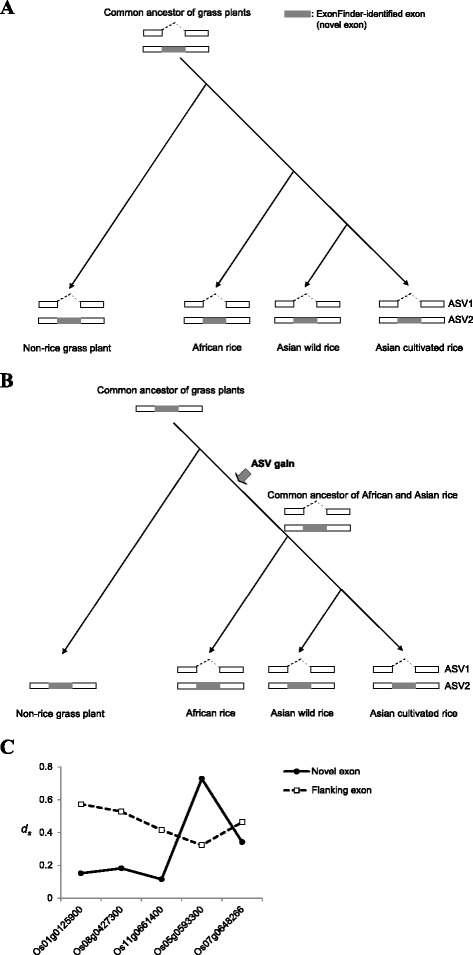
**Possible evolutionary scenarios of the previously-annotated isoforms that exclude the novel exons (ASV1) and the newly-identified isoforms that include the novel exons (ASV2) during the evolution of rice transcriptome. (A)** Both isoforms (ASV1 and ASV2) might have been present in the common ancestral transcriptome of grass plants. **(B)** A gain-of-ASV event might occur after the divergence of rice and non-rice plants. **(C)** Comparison of *ds* values of novel exons and their corresponding flanking exons.

According to our experimental validation, there were six genes (i.e., Os08g0427300, Os01g0125900, Os05g0593300, Os11g0661400, Os07g0648266, and Os04g0582600) in which the previously-annotated isoforms that exclude the novel exons (designated as “ASV1”) and newly-identified isoforms that include the novel exons (designated as “ASV2”) were steadily detected in all rice species examined (Figure 4). Since both ASV1 and ASV2 were detected in Asian cultivated/wild rice and African cultivated rice, we hypothesized that both isoforms for each of the six genes might have been present in the common ancestral transcriptome of African and Asian rice species. Moreover, since the novel exons were derived from comparative analysis of non-rice EST traces, we speculated that ASV2 might also represent a common ancestral isoform of grass plants. As for ASV1, there are two possible scenarios. First, both ASV1 and ASV2 might be present in the common ancestral transcriptome of grass plants, inferring that the novel exons exhibited alternatively spliced exons (ASEs) in both rice and other grass plants (designated as “conserved ASEs”) (Figure 5A). This implies that both AS isoforms are functionally important across grass plants. Second, ASV1 might represent a gain-of-ASV event that occurred after the divergence between rice and non-rice plants, inferring that the novel exons were constitutively spliced exons (CSEs) in the common ancestral transcriptome of grass plants (designated as “lineage-specific ASEs”) (Figure 5B). This implies that ASV1 may play a lineage-specific role in rice. Our previous study has showed that the *ds* values of conserved ASEs were markedly lower than those of both lineage-specific ASEs and CSEs [40], providing a possible way to examine whether the novel exons are conserved ASEs. To this end, on the basis of the rice-maize-sorghum orthologues (Additional file 3) and the phylogenetic context of these three species, we calculated the evolutionary rates of the rice transcript sequences and their orthologous sequences derived from the rice-maize-sorghum common ancestor using the CodeML program of PAML [60,61]. As shown in Figure 5C, the *ds* values of the novel exons were lower by three-fold or more compared with those of their flanking exons for Os08g0427300, Os01g0125900, and Os11g0661400, suggesting that the novel exons were subjected to be alternatively spliced in the rice-maize-sorghum common ancestral transcriptome. Meanwhile, for Os05g0593300 and Os07g0648266, the *ds* values of the novel exons were greater or insignificantly lower than those of their flanking exons (Figure 5C), inferring that the novel exons might be lineage- or rice-specific ASEs. Of note, Os04g0582600 was not considered due to the lack of the information of orthologues. We further aligned ASV1/ASV2 against currently-available non-rice transcripts and found that non-rice transcript evidence supported both ASV1 and ASV2 in Os08g0427300, Os01g0125900, and Os11g0661400, while non-rice evidence only supported ASV2 in Os05g0593300 and Os07g0648266 (Additional file 4). This result also supported the above speculation.

In summary, the above examples illustrate that the identified ASVs can serve a source for inferring the ancestral transcriptomes of rice and other grass plants. If the newly-identified ASVs (ASV2) were not considered in either of the above scenarios, one might speculate that ASV2 had been lost in rice, and the interpretation of transcriptome evolution could be incomplete or even misleading. The ASVs that were inferred from such a comparative analysis of cross-species EST library therefore provide new insights into evolutionary transcriptomic studies.

### Implications of distinct ASVs for analysis of expression divergence

**Figure 6:**
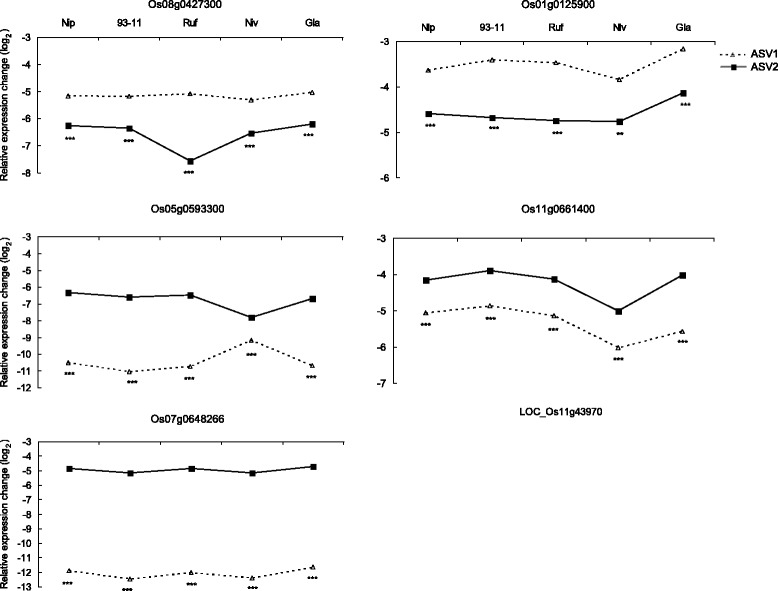
**The qRT-PCR expression analysis of the previously-annotated isoforms that exclude the novel exons (ASV1) and the newly-identified isoforms that include the novel exons (ASV2) in the rice cultivars and wild species.** Statistical significance was estimated by the two-sided *t*-test. **P < 0.01 and ***P < 0.001.

We then probed expression divergence of distinct ASVs (i.e., ASV1 and ASV2) among the five rice species examined. We analyzed the expression profiles of ASV1 and ASV2 for Os08g0427300, Os01g0125900, Os05g0593300, Os11g0661400, and Os07g0648266 by qRT-PCR (Figure 6). Of note, Os04g0582600 was not considered here because of difficulties in generating suitable primers for qRT-PCR. Two intriguing observations were made. First, ASV1 and ASV2 exhibited significantly different expression levels for all five genes in all rice species examined (all *P* values < 0.01 by the two-tailed *t*-test; Figure 6), suggesting that these two distinct AS isoforms might play different functional roles. Importantly, for Os05g0593300, Os11g0661400, and Os07g0648266, the expression levels of ASV2 were remarkably higher than those of ASV1 in all rice species examined, indicating that the newly-identified isoforms (i.e., ASV2) predominated over their previously-annotated counterparts (i.e., ASV1) for these genes. Second, the trend that ASV1 was more highly expressed than ASV2 for Os08g0427300 and Os01g0125900 but the reverse was true for Os05g0593300, Os11g0661400, and Os07g0648266 was observed in all five rice species examined (Figure 6). These results suggested that such ASV1 and ASV2 expression profiles for the five genes were present in the ancestral transcriptome before the domestication of Asian/African rice. Since *O. sativa* (such as *japonica* and *indica*; two Asian rice cultivars) and *O. glaberrima* (an African cultivated rice species) have independent histories of domestication [62,63], maintenance of such expression profiles may be of great importance during the domestication and evolution of rice transcriptome.

## Discussion

In this study, we described an *in silico* pipeline ExonFinder to identify novel exons/ASVs based on comparative analysis of cross-species EST library. Using ExonFinder we identified 2,963 ASVs with 2,879 novel exons (including cassette exons and retained introns) that were previously unannotated in rice, maize, and sorghum. RT-PCR-sequencing confirmed the authenticity of 81% of the tested ASVs, supporting the effectiveness of the ExonFinder pipeline. Cross-species conservation of these exons/ASVs implies their biological importance and functional properties. In addition, a considerable proportion of newly-identified exons have no significant difference in evolutionary rates as compared to their flanking exons, suggesting that these novel exons and their flanking partners tend to co-occur in the transcripts (Figure 3B). While 13 novel ASVs were experimentally validated, 54% of their corresponding previously-annotated ASVs were not detected (Figure 4A and B). Such results were consistent across multiple rice species including cultivated and wild rice species (Figure 4A and B). This reveals that some of the previous annotations might be subject to erroneous prediction. These observations also indicate the capability and usefulness of ExonFinder for the curation and improvement of current plant genome annotations.

Regarding AS patterns, intron-retention events were observed to be the most prevalent AS event in plants such as rice and *Arabidopsis*, contributing to a higher proportion of all ASVs than cassette exons [10,14,26]. However, ExonFinder identified fewer retained introns than cassette exons (Table 2). There are several possibilities. First, the majority of retained introns are subject to nonsense-mediated mRNA decay [26,64], which tend to locate at the 3′ terminal of transcripts. ExonFinder was designed for the accurate identification of internal exons, resulting in the limitation for the detection of exons close to the terminal of transcripts. This property also accounts for the observation that the number of UTR exons identified by ExonFinder is smaller than that of CDS exons (Table 2). Second, most of the retained introns in rice were supported by rice EST traces, and therefore were removed during the examination of exon novelty. In fact, before this examination, the number of identified retained introns (1,040) was much larger than that of identified cassette exons (533) (Additional file 5). Third, a substantial number of retained introns may be derived from spurious EST traces, such as genomic DNA contamination and incompletely-processed transcripts [26].

ExonFinder has the capability to identify “full-length” novel exons with cross-species EST evidence. For novel cassette exons, each identified ASV must have at least one EST from other species to support the corresponding exon-intron boundaries and two previously-annotated flanking exons (Figure 1B). For novel retained introns, ExonFinder requires EST evidence from other species to support the corresponding region along with the flanking exons (Figure 1B). We also demonstrated that ExonFinder is capable of inferring gain- and loss-of-ASV events, which may be valuable for evolutionary studies in the era of transcriptomics. Although the lack of an adequate quantity of EST traces in many plant species may hamper such a comparative transcriptome analysis, the rapid progress in high-throughput RNA sequencing (RNA-seq) technologies can provide alternative resources for this task. For example, transcript assemblers (e.g., Cufflinks [65] and Trinity [66]) can subsequently be used to generate preliminary multiple-exon transcript segments based on integration of long (e.g., PacBio-based) and short (e.g., Illumina-based) RNA-seq reads [67]. In this regard, ExonFinder can make a knowledge-based complement to these strategies, by revising the transcriptome annotation through the lens of cross-species comparative analysis.

For accuracy, ExonFinder does not consider the candidates that change the reading frame of the corresponding transcript or result in premature stop codons; however, some of these exons may be fragments of non-coding RNAs that are expressed in the target species. For example, we showed an exon candidate that resulted in a premature stop codon was experimentally-validated in multiple rice cultivars and wild species (Additional file 6), suggesting that this exon might be of biological significance in rice transcriptome. Thus, it is also possible to utilize ExonFinder to identify non-coding RNAs with cross-species conservations. Moreover, we showed that evolutionary rates might serve as an indicator for determining whether the identified exons were alternatively spliced. Integration of comparative analysis (ExonFinder) and evolutionary analysis (evolutionary rates) may enable the accurate identification of novel AS events by using publicly-available EST traces, without requiring costly experiments. In conclusion, this study not only presents an *in silico* pipeline for accurate identification of novel exons/ASVs, but also expands the discovery of AS events in the evolution of plant transcriptomes.

## Conclusions

We have described a computational pipeline (ExonFinder) to identify previously-unannotated exons and ASVs using cross-species EST-to-genome comparisons. RT-PCR-sequencing confirmed 81% of the tested exons/ASVs, supporting the effectiveness of our *in silico* strategy. Exonfinder thus reannotated the rice, maize, and sorghum genomes, and identified many novel exons/ASVs that are cross-species conserved in grass plant ESTs. Evolutionary analysis further revealed the consistency of evolutionary rates between certain novel exons and their flanking exons, which provided evidence of their co-occurrence in the transcripts, suggesting that previously-annotated isoforms might be subject to erroneous predictions. This also indicated that evolutionary rates might serve as an indicator for determining whether the identified exons were alternatively spliced. Moreover, comparative analyses inferred the common ancestral transcriptome of grass plants and gain- and loss-of-ASV events, providing important targets for evolutionary and functional studies. Exonfinder can be applied to comparative analysis of other model organisms.

## Methods

### Data retrieval and availability

Genomic sequences and gene annotations of rice (*Oryza sativa*), maize (*Zea mays*), and sorghum (*Sorghum bicolor*) were downloaded from the EnsemblPlants genome browser at http://plants.ensembl.org (release 18). Of note, for rice, EnsemblPlants provided annotations from both the MSU Rice Genome Annotation Project and the Rice Annotation Project Database (RAP-DB)/International Rice Genome Sequencing Project. EST traces of nine grass species: rice, maize, sorghum, barley (*Hordeum vulgare*), meadow ryegrass (*Festuca pratensis*), purple false brome (*Brachypodium distachyon*), sugarcane (*Saccharum officinarum*), switchgrass (*Panicum virgatum*), and wheat (*Triticum aestivum*) were downloaded from the repository of NCBI UniGene Database at ftp://ftp.ncbi.nih.gov/repository/UniGene/ (Table 1).

### The *in silico* pipeline of ExonFinder

ExonFinder was designed to identify novel exons in a target species on the basis of comparative analysis of the public EST library from one or more non-target species (subject species). As shown in Figure 1A, we first used BLAT [68] to align EST traces from subject species (see Table 1) against the genome of the target species. We considered the best hit in the case of multiple matches. We then extracted the genomic sequences of the target species that were previously annotated as introns and conserved in subject species ESTs, which represented the candidates of novel exons/ASVs. As shown in Figure 1B, two ASV types were identified: retained introns and cassette exons. Taking rice for example, we found approximately 2.5 million non-rice EST matches that located within introns and without overlapping with any annotated transcript (Figure 1). To guarantee the accuracy and novelty of the identified exons, we only considered the exons that satisfied the following four criteria: (1) the length of an exon candidate should be longer than 50 bp (in the case of cassette exons) and the lengths of both flanking EST segments of the exon candidate should overlap with the corresponding previously-annotated transcript by ≥ 50 bp; (2) a candidate of novel cassette exon must be flanked by canonical splicing sites (i.e., GT-AG, GC-AG, or AT-AC [69]) within a 10-bp window at boundaries of the candidate; non-canonical splicing sites (i.e., AT-AA, AT-AG, AT-AT, GT-AT, or GT-GG [69]) were considered, if the above-mentioned canonical splicing sites can not be detected within such a window; (3) an exon candidate that located within CDS must not alter the reading frame of the corresponding previously-annotated transcript and must not result in any premature stop codon; and (4) an exon candidate should not overlap with any EST trace of the target species or known transcripts from the Ensembl annotation. With these criteria, the number of candidates was largely reduced to several hundreds. All the programs of the ExonFinder pipeline were implemented in C language under Linux environment (i.e., Bio-Linux 6). Of note, ExonFinder also identifies novel exons/ASVs with EST evidence from the same species; users can choose whether or not to remain this information.

### Comparison of *dn* and *ds* values between novel exons and their flanking exons

For each newly-identified ASV that located in CDS region and was supported by EST traces from more than one subject species, we selected the best hit of the matched EST traces. The two flanking CDS exons of the novel exon were concatenated. The reading frames of the novel exon and the concatenated sequence were determined according to the Ensembl annotation of the target species. Subsequently, the numbers of synonymous and non-synonymous sites, and the *dn*, *ds*, and *dn/ds* values of the novel exon and the concatenated sequence were respectively calculated using the YN00 program of the PAML package [60,61]. For detecting significant differences in *dn* values between the novel exon and the concatenated sequence (i.e., the flanking exons of the novel exon), we created a two-way contingency table, with rows comprised of the numbers of nonsynonymous sites of the novel exon and the concatenated sequence, and columns comprised of the numbers of changed and unchanged sites; this table was used to test the independence of the number of changed nonsynonymous sites between the novel exon and the concatenated sequence using the two-tailed Fisher’s exact test. The similar processes were applied to detecting significant differences in *ds* values between the novel exon and the concatenated sequence.

### Rice material and growth conditions

Three rice cultivars (*O. sativa* L. ssp. *japonica* cv. Nipponbare, *O. sativa* L. ssp. *indica* cv. 93-11, and *O. glaberrima* [IRGC accession number 96717]) and two wild species (*O. rufipogon* [IRGC accession number 105491] and *O. nivara* [IRGC accession number 100897]) were used in this study. Seeds of each species were sterilized with 2.5% sodium hypochlorite for 20 minutes, and were cleaned with distilled water. Germination of seeds was triggered in petri dishes with wet papers at 37°C in the dark for two days, and then the germinated seeds were moved to culture in beakers (600 mL) containing half-strength Kimura B nutrient solution. Hydroponic cultivation was performed under natural sunlight at 30/25°C day/night and 70% relative humidity in the greenhouse of the Genomics Research Center, Academia Sinica, Taiwan. Nutrient solutions (pH 4.7-4.8) were replaced every three days. Once the third leaf was fully extended, the seedling was immediately collected for use in experiments.

### RT-PCR screening

Total RNA was isolated from seedlings using TRIzol reagent (Invitrogen, CA, USA) according to the manufacturer’s instructions. RNA was subsequently treated with Turbo DNase I (Ambion, TX, USA) to remove contamination of genomic DNA. For first strand cDNA synthesis, 200 ng total RNA was reverse transcribed with SuperScript II enzyme (Invitrogen, CA, USA). The primer pairs for novel exon screening were designed against the two flanking exons of each exon identified by ExonFinder (Additional file 7). The RT-PCR thermal cycle was as follows: denaturation at 95°C for 5 minutes; 40 cycles of 95°C for 30 seconds, 58°C for 30 seconds, and 72°C for 40 seconds; final extension at 72°C for 7 minutes; and final cooling to 16°C. The amplicons were separated by electrophoresis in a 2% agarose gel, and were visualized with ethidium bromide staining. Images of amplicons were captured using an E-box Vx2 system (Vilber Lourmat, Germany).

### Quantitative RT-PCR (qRT-PCR) analysis

To quantify the expression of validated novel exons in rice species, primer pairs specifically targeting well-annotated and novel transcripts were designed (Additional file 8). Quantitative RT-PCR analysis was performed with a KAPA SYBR FAST qPCR Kit (KAPA Biosystems, USA) and a Roche LightCycler 480 Real-Time PCR System (Roche Diagnostics, USA). The amplification program consisted of the following steps: pre-incubation at 95°C for 3 minutes; 40 cycles of amplification (95°C for 10 seconds, 60°C for 20 seconds, and 72°C for 1 second); and melting curve analysis (95°C for 5 seconds, 65°C for 1 minute, and finally 5 - 10 continuous acquisitions to 97°C). The amplicons were further validated by electrophoresis and sequencing. Levels of each gene were normalized to the internal control OsUbiquitin (GENBANK/D12629); each result was shown as the mean of four independent experiments.

### Availability of supporting data

The package of ExonFinder along with documentation, and the full list of novel exons/ASVs identified in this study are publicly available at http://exonfinder.sourceforge.net/ and Additional file 1, respectively. The multiple alignments of rice-maize-sorghum orthologous sequences of the ExonFinder-identified exons and their concatenated flanking exons for Os08g0427300, Os01g0125900, Os05g0593300, Os11g0661400, and Os07g0648266 are available in Additional file 3. The RT-PCR and Q-RT-PCR primer pairs used in this study are listed in Additional files 7 and 8, respectively.

## Additional files

Additional file 1:
**The full list of novel rice, maize, and sorghum exons/ASVs identified in this study.**
Additional file 2:
**Experimental validation and evolutionary examination of the previously-annotated isoforms that exclude the novel exons and the newly-identified isoforms that include the novel exons.**
Additional file 3:
**The multiple alignments of rice-maize-sorghum orthologous sequences of novel exons and their concatenated flanking exons for Os08g0427300, Os01g0125900, Os05g0593300, Os11g0661400, and Os07g0648266.**
Additional file 4:
**Non-rice EST evidence of the ASVs that include (novel ASVs) or exclude (annotated ASVs) the novel exons within the six genes: Os08g0427300, Os01g0125900, Os05g0593300, Os11g0661400, Os07g0648266, and Os04g0582600.**
Additional file 5:
**Number of identified rice exons/ASVs without filtering out the exons/ASVs supported by rice EST traces.**
Additional file 6:
**(A) Gene structure and (B) RT-PCR products of the ExonFinder-extracted exon containing a premature stop codon within Os01g0692300.** Ruf, *O. rufipogon*; Niv, *O. nivara*; Nip, *O. sativa* L. ssp. *japonica* cv. Nipponbare; 93-11, *O. sativa* L. ssp. *indica* cv. 93-11; Gla, *O. glaberrima*. Additional file 7:
**RT-PCR primer pairs used in this study.**
Additional file 8:
**Q-RT-PCR primer pairs used in this study.**

## Abbreviations

AS: Alternative splicing
ASV: Alternatively spliced variant
EST: Expressed sequence tag
UTR: Untranslated region
CDS: Coding sequence
*dn*: non-synonymous substitution rate
*ds*: synonymous substitution rate
Nip: *Oryza sativa* L. ssp. *japonica* cv. Nipponbare
93-11: *O. sativa* L. ssp. *indica* cv. 93-11
Gla: *O. glaberrima*
Ruf: *O. rufipogon*
Niv: *O. nivara*
Os: rice
Fp: Meadow ryegrass
Ta: wheat
Hv: Barley
Bd: Purple false brome
Sof: Sugarcane
Sb: Sorghum
Zm: Maize
Pv: switchgrass
