# Supplementary material for: Comparative genomics of grass EST libraries reveals previously uncharacterized splicing events in crop plants
Source: BMC Plant Biol. 2015 Feb 5;15:39. doi: 10.1186/s12870-015-0431-7 (PMC4323234; doi:10.1186/s12870-015-0431-7)
Supplement: Additional file 2: — Experimental validation and evolutionary examination of the previously-annotated isoforms that exclude the novel exons and the newly-identified isoforms that include the novel exons. [file 12870_2015_431_MOESM2_ESM.doc]

**Additional file 2.** Experimental validation and evolutionary examination of the previously-annotated isoforms that exclude the novel exons and the newly-identified isoforms that include the novel exons.

| **Gene ID** | **Genomic type of the novel exon** | **Gene structures of the previous-annotated (i.e., Ensembl-annotated) and novel forms** | **Experimentally- validated forms** | **Novel_*dn* > Flanking_*dn* or Novel_*ds* < Flanking_*ds*?** |
| --- | --- | --- | --- | --- |
| Os08g0427300 | CDS | 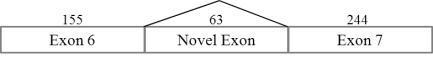 | Both forms | Yes (significant^*^) |
| Os01g0125900 | CDS | 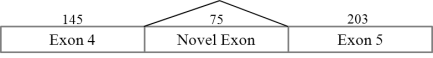 | Both forms | Yes (significant^*^) |
| Os05g0593300 | CDS | 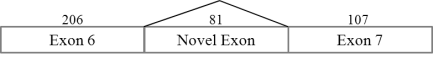 | Both forms | Yes (significant^*^) |
| Os11g0661400 | CDS | 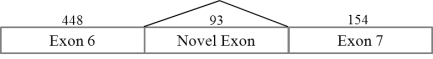 | Both forms | Yes (significant^*^) |
| Os07g0648266 | CDS | 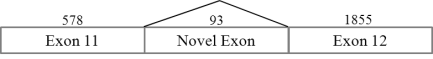 | Both forms | Yes |
| Os04g0582600 | CDS | 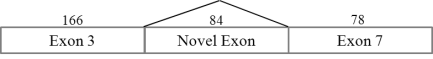 | Both forms | Yes |
| Os07g0497000 | CDS | 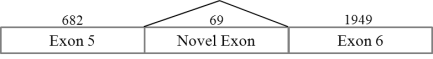 | Novel form only | Yes |
| Os01g0388500 | CDS | 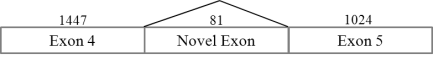 | Novel form only | Yes |
| Os02g0137450 (Os02g0137500) | CDS | 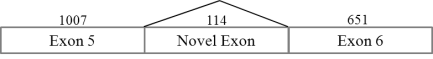 | Novel form only | No |
| Os11g0244300 | CDS | 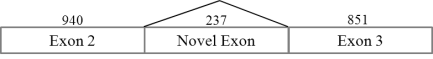 | Novel form only | Yes (significant^*^) |
| Os01g0695800 | CDS | 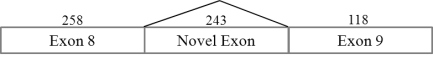 | Novel form only | Yes |
| Os02g0114000 | CDS | 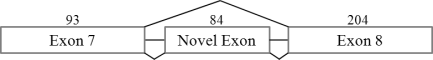 | Novel form only | No |
| Os06g0472300 | 5’UTR | 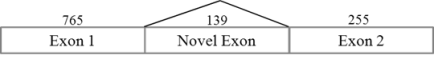 | Novel form only | - |
| Os02g0605600 | CDS | 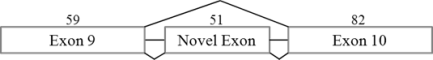 | None | - |
| Os04g0353000 | CDS | 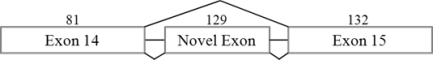 | None | - |
| Os11g0543800 | CDS | 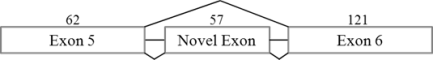 | None | - |

^*^ The novel exons have significantly higher *dn* values or significantly lower *ds* values than their corresponding flanking exons (*P* value < 0.05 by the two-tailed Fisher's Exact test, see Materials and Methods).
