# Supplementary material for: Comparative genomics of grass EST libraries reveals previously uncharacterized splicing events in crop plants
Source: BMC Plant Biol. 2015 Feb 5;15:39. doi: 10.1186/s12870-015-0431-7 (PMC4323234; doi:10.1186/s12870-015-0431-7)
Supplement: Additional file 4: — Non-rice EST evidence of the ASVs that include (novel ASVs) or exclude (annotated ASVs) the novel exons within the six genes: Os08g0427300, Os01g0125900, Os05g0593300, Os11g0661400, Os07g0648266, and Os04g0582600. [file 12870_2015_431_MOESM4_ESM.doc]

**Additional file 4.** Non-rice transcript evidence of the ASVs that include (novel ASVs) or exclude (annotated ASVs) the novel exons within the five genes: Os08g0427300, Os01g0125900, Os05g0593300, Os11g0661400, and Os07g0648266.


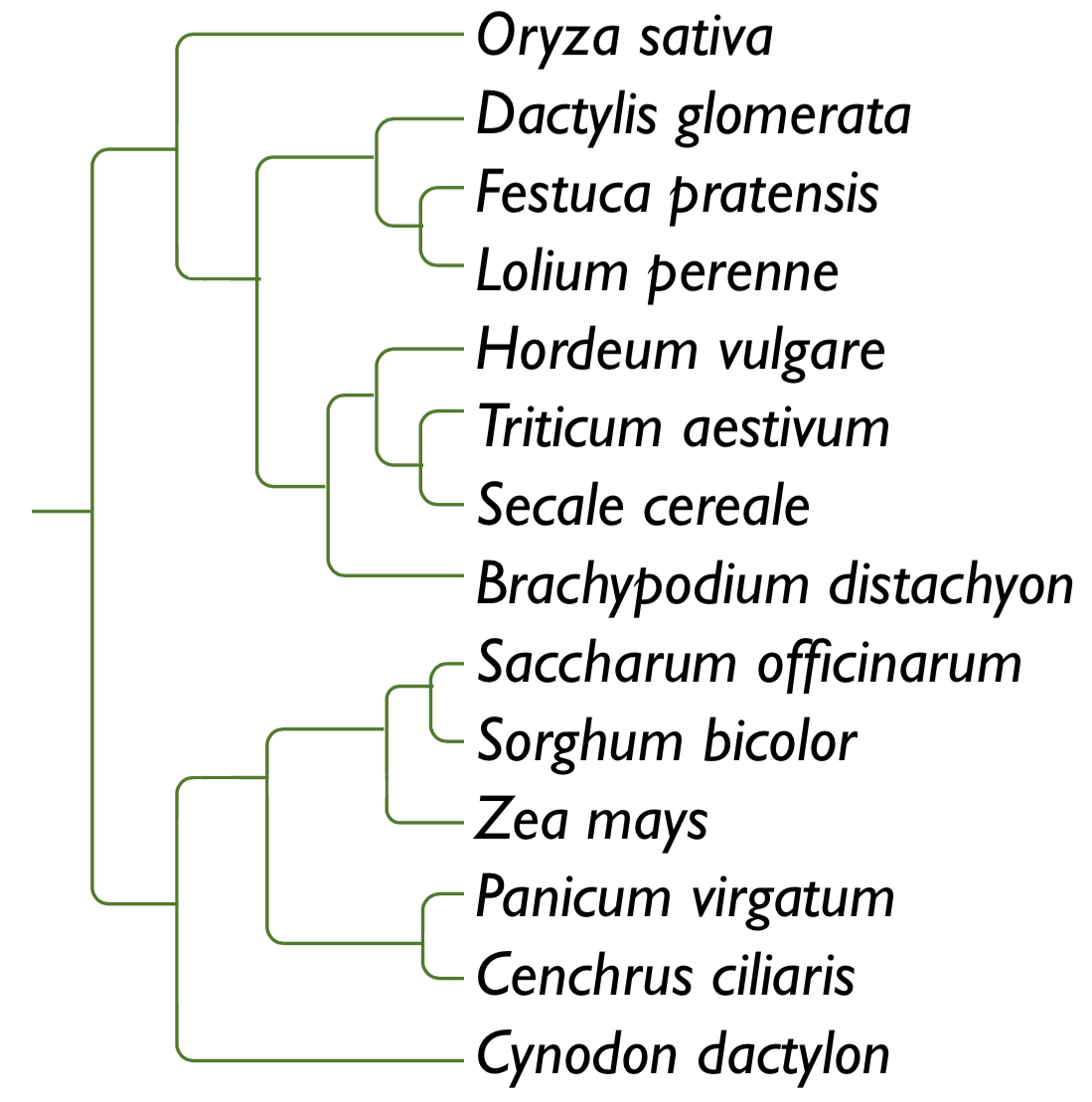

Note: Non-rice grass transcript evidence was inferred from the MSU genome browser [1] at http://rice.plantbiology.msu.edu/cgi-bin/gbrowse/rice/ and the EST traces of the grass species examined (see Table 1). The phylogenetic tree of rice and the non-rice grass plants examined [2-3] was also provided.

**References**

1. Kawahara Y, de la Bastide M, Hamilton JP, Kanamori H, McCombie WR, Ouyang S, Schwartz DC, Tanaka T, Wu J, Zhou S *et al*: **Improvement of the Oryza sativa Nipponbare reference genome using next generation sequence and optical map data**. *Rice (N Y)* 2013, **6**(1):4.

2. Kellogg EA: **Evolutionary history of the grasses**. *Plant Physiol* 2001, **125**(3):1198-1205.

3. Bouchenak-Khelladi Y, Salamin N, Savolainen V, Forest F, Bank M, Chase MW, Hodkinson TR: **Large multi-gene phylogenetic trees of the grasses (Poaceae): progress towards complete tribal and generic level sampling**. *Mol Phylogen Evol* 2008, **47**(2):488-505.
