# Supplementary material for: Comparative genomics of grass EST libraries reveals previously uncharacterized splicing events in crop plants
Source: BMC Plant Biol. 2015 Feb 5;15:39. doi: 10.1186/s12870-015-0431-7 (PMC4323234; doi:10.1186/s12870-015-0431-7)
Supplement: Additional file 5: — Number of identified rice exons/ASVs without filtering out the exons/ASVs supported by rice EST traces. [file 12870_2015_431_MOESM5_ESM.doc]

**Additional file 5.** Summary of identified rice exons/ASVs without filtering out the exons/ASVs supported by rice EST traces.

|  | **Newly-identified exons (ASVs)** | | |
| --- | --- | --- | --- |
| **Genomic Type** | **Cassette** | **Retained intron** | **Total** |
| **5'-UTR** | 77 (99) | 377 (415) | 454 (514) |
| **CDS** | 390 (378) | 264 (268) | 654 (646) |
| **3'-UTR** | 66 (61) | 399 (463) | 465 (524) |
| **Total** | 533 (538) | 1040 (1146) | 1573 (1684) |
