# Supplementary material for: Comparative genomics of grass EST libraries reveals previously uncharacterized splicing events in crop plants
Source: BMC Plant Biol. 2015 Feb 5;15:39. doi: 10.1186/s12870-015-0431-7 (PMC4323234; doi:10.1186/s12870-015-0431-7)
Supplement: Additional file 6: — (A) Gene structure and (B) RT-PCR products of the ExonFinder-extracted exon containing a premature stop codon within Os01g0692300. Ruf, O. rufipogon; Niv, O. nivara; Nip, O. sativa L. ssp. japonica cv. Nipponbare; 93-11, O. sativa L. ssp. indica cv. 93-11; Gla, O. glaberrima. [file 12870_2015_431_MOESM6_ESM.doc]

**(A)**

**(B)**

**Additional file 6.** (A) Gene structure and (B) RT-PCR products of an identified exon containing a premature stop codon within Os01g0692300. Ruf, *O. rufipogon*; Niv, *O. nivara*; Nip, *O. sativa* L. ssp. *japonica* cv. Nipponbare; 93-11, *O. sativa* L. ssp. *indica* cv. 93-11; Gla, *O. glaberrima*.
