# Supplementary material for: Comparative genomics of grass EST libraries reveals previously uncharacterized splicing events in crop plants
Source: BMC Plant Biol. 2015 Feb 5;15:39. doi: 10.1186/s12870-015-0431-7 (PMC4323234; doi:10.1186/s12870-015-0431-7)
Supplement: Additional file 7: — RT-PCR primer pairs used in this study. [file 12870_2015_431_MOESM7_ESM.doc]

**Additional file 7.** RT-PCR primer pairs used in this study.

| Gene name | Primer ID | Primer sequence |
| --- | --- | --- |
| Os08g0427300 | I-01-Fw | CTTGAGGTTAACTCCTCGTG |
|  | I-01-Rv | TCTGAAGTAGTGGAACCAGC |
| Os01g0125900 | I-04-Fw | TGACTCGGCTCGCGAGTGAA |
|  | I-04-Rv | CGGTACTCTCAGATGTTGCC |
| Os05g0593300 | I-05-Fw | GTTCTGTTCCAGGAGCATT |
|  | I-05-Rv | GATTATGTGGCCGTAAGGT |
| Os04g0582600 | I-06-Fw | GTCCAATACAGGATGCATCC |
|  | I-06-Rv | CAGGCTACGACCATTCTTCT |
| Os11g0661400 | I-09-Fw | TCTGATGCTCATCTTGGTGC |
|  | I-09-Rv | CTTAAAGGCTTGGCATCTGC |
| Os07g0648266 | I-10-Fw | CTAGTCTAGATGGAGACCCT |
|  | I-10-Rv | TGAAGACGCTTCAACAGCCA |
| Os07g0497000 | I-07-Fw | CAGCTGGATTTCTGAATCTG |
|  | I-07-Rv | CACGTGCATTCGTCATATGG |
| Os01g0388500 | I-08-Fw | TCGTCTGCAAACATGCCGAT |
|  | I-08-Rv | TTCAGCTCAGTGTAGATCAG |
| Os02g0137450 | I-11-Fw | ACTGTGACTCCAGCTGATCC |
|  | I-11-Rv | AGTTCAGCTGAACGCTGCAG |
| Os11g0244300 | I-13-Fw | TCTTGCACTCCAGTCTCCAC |
|  | I-13-Rv | TTCGGCCATCCAATCTGCTC |
| Os01g0695800 | I-14-Fw | CCTTCAAGAGCCCCTTAGGA |
|  | I-14-Rv | GCAAGCAACCTCTTGGTGCA |
| Os02g0605600 | S-01-Fw | GCGCCTGTGTGACATATGTG |
|  | S-01-Rv | CTGCAGTTCGGATAACTTCT |
| Os11g0543800 | S-05-Fw | GCAGAAGGGAGTGTTCAAGC |
|  | S-05-Rv | TGAGCACGAAAAAGAAGTGC |
| Os02g0114000 | S-08-Fw | GTACCGAATGCTGTCTTGGT |
|  | S-08-Rv | CCGTTGGGCCTCATCAATAA |
| Os02g0247000 | S-09-Fw | AGGTCGTGGAAGTACCGTCA |
|  | S-09-Rv | CCATGGAAAGAAGCCCAGTA |
| Os04g0353000 | S-12-Fw | GCCGAAGTATGTCACCGTTT |
|  | S-12-Rv | CAACATGGCCAGCTTAGTGA |
