# Supplementary material for: Comparative genomics of grass EST libraries reveals previously uncharacterized splicing events in crop plants
Source: BMC Plant Biol. 2015 Feb 5;15:39. doi: 10.1186/s12870-015-0431-7 (PMC4323234; doi:10.1186/s12870-015-0431-7)
Supplement: Additional file 8: — Q-RT-PCR primer pairs used in this study. [file 12870_2015_431_MOESM8_ESM.doc]

**Additional file 8.** qRT-PCR primer pairs used in this study.

| Gene name | Isoform | Primer ID | Primer sequence |
| --- | --- | --- | --- |
| Os08g0427300 | Annotated form | q-I-01W-Fw | CTTCAGTAGAAAGGCCTCTC |
|  |  | q-I-01W-Rv | TCTGAAGTAGTGGAACCAGC |
|  | Novel form | q-I-01N-Fw | CAGGGATTGCAGGAGGTCAA |
|  |  | q-I-01N-Rv | TCTGAAGTAGTGGAACCAGC |
| Os01g0125900 | Annotated form | q-I-04W-Fw | TGACTCGGCTCGCGAGTGAA |
|  |  | q-I-04W-Rv | TCAACATCTACAGACTTCCC |
|  | Novel form | q-I-04N-Fw | GATGGATCAAACTCAGATGC |
|  |  | q-I-04N-Rv | CGGTACTCTCAGATGTTGCC |
| Os05g0593300 | Annotated form | q-I-05W-Fw | AGGACTAAATCGAGCCAACA |
|  |  | q-I-05W-Rv | GATTATGTGGCCGTAAGGT |
|  | Novel form | q-I-05N-Fw | GTTCTGTTCCAGGAGCATT |
|  |  | q-I-05N-Rv | AGCCTACAGTTGCAGCAGGC |
| Os04g0582600 | Annotated form | q-I-06W-Fw | GGGCTACAGTAAGCACCTTC |
|  |  | q-I-06W-Rv | GACCATTCTTCTCAGTAGTG |
|  | Novel form | q-I-06N-Fw | GGGCTACAGTAAGCACCTTC |
|  |  | q-I-06N-Rv | GACCCCCATCTACATTAATA |
| Os11g0661400 | Annotated form | q-I-09W-Fw | TTGGTGCTGAAATCGCTCTT |
|  |  | q-I-09W-Rv | CTTAAAGGCTTGGCATCTGC |
|  | Novel form | q-I-09N-Fw | GGCCAGTTCTGAGGATGATTGC |
|  |  | q-I-09N-Rv | CTTAAAGGCTTGGCATCTGC |
| Os07g0648266 | Annotated form | q-I-10W-Fw | CTAGTCTAGATGGAGACCCT |
|  |  | q-I-10W-Rv | CCCAAGAGCATCGGCCGAAA |
|  | Novel form | q-I-10N-Fw | CTAGTCTAGATGGAGACCCT |
|  |  | q-I-10N-Rv | GGACATGCAAAATGTGCCGA |
